# Comparison of blood lipid profile/thyroid function markers between unipolar and bipolar depressed patients and in depressed patients with anhedonia or suicidal thoughts

**DOI:** 10.1186/s10020-019-0119-9

**Published:** 2019-11-20

**Authors:** Meilei Su, Enze Li, Chong Tang, Yongzhi Zhao, Ruqing Liu, Keming Gao

**Affiliations:** 1 Department of Psychiatry, Nanfang Hospital, Southern Medical University, 1838 North Guangzhou Avenue, Guangzhou, China; 2 Guangzhou Key Laboratory of Environmental Pollution and Health Risk Assessment, Department of Preventive Medicine, School of Public Health, Sun Yat-sen University, Guangzhou, China; 3 Mood and Anxiety Clinic in the Mood Disorders Program, Department of Psychiatry, Case Western Reserve University School of Medicine/University Hospitals Cleveland Medical Center, Cleveland, OH USA

**Keywords:** Unipolar depression, Bipolar depression, Lipid profile, Anhedonia, Suicidal thoughts

## Abstract

**Background:**

This study aimed to investigate the differences in the serum levels of glucose, lipid, and thyroid function markers between unipolar and bipolar depressed patients, as well as the effect of anhedonia and suicidal thoughts on the levels of these biochemical parameters.

**Methods:**

A total of 287 unmedicated depressed patients from January 2016 to December 2017 were included in this study, including 92 bipolar depressions and 195 unipolar depressions. Anhedonia was determined using the item 32 of Symptom Checklist (SCL-90). Suicide ideation was assessed by item 15 of SCL-90.

**Results:**

The bipolar group had significantly lower lipid levels (including triglycerides, cholesterol, low-density lipoprotein cholesterol [LDL], very low-density lipoprotein cholesterol [VLDL]) and insulin resistance index but higher levels of prolactin, low triiodothyronine (T3) and free T3 (FT3) as well as higher incidence of anhedonia as compared with the unipolar group. Depressed patients with anhedonia had significantly higher LDL level than those without anhedonia. Depressed patients with suicidal thoughts had cholesterol and high-density lipoprotein cholesterol (HDL) level. The above-mentioned differences were confirmed by logistic regression analysis. Receiver operating characteristic curve (ROC) analysis showed that the area under the ROC curve (AUC) ranged from 0.546 to 0.685.

**Conclusion:**

Triglycerides, cholesterol, LDL, VLDL T3, FT3 levels were significantly different between unipolar and bipolar depressed patients, which might have the potential to be the markers for differential diagnosis. Patients with anhedonia had lower LDL level, while patients with suicidal thoughts had higher levels of cholesterol and HDL as compared with the corresponding control groups.

## Background

The link between serum lipid level and depression has been reported over the past three decades (Hawton et al., 1993). A number of previous studies have shown that lower cholesterol level is associated with depression (Morgan et al., 1993; Olusi & Fido, 1996; Ong et al., 2016; Tedders et al., 2011). Individuals with low cholesterol level have an elevated risk of depression (Steegmans et al., 1996) and suicide risk (Kunugi et al., 1997; Wu et al., 2016). Maes et al. show that lower high-density lipoprotein cholesterol (HDL) is a marker for major depression and suicidal behavior in depressed patients (Maes et al., 1997). A meta-analysis including 510,392 subjects from 65 studies demonstrates that subjects with suicidal thoughts had significantly lower levels of serum total cholesterol, low-density lipoprotein cholesterol (LDL), and triglycerides than the non-suicidal subjects (Wu et al., 2016). Likewise, van Reedt Dortland et al. report that melancholic features were independently associated with HDL (van Reedt Dortland et al., 2010). Ainiyet et al. have reported that suicidal thoughts are associated with low total cholesterol, LDL, and total lipids in both unipolar and bipolar depressed patients (Ainiyet & Rybakowski, 2014). The mechanism underlying the association between serum lipids and suicidality is still unclear. One possible explanation is that individuals with suicidal behavior may have depressive episodes that influenced their appetites and caused weight loss and, in turn, resulted in lower serum lipid levels (Zhang et al., 2005). Another explanation is that low cholesterol concentration and suicidality are connected with T cells-produced interleukin-2, causing a decrease in serum cholesterol and an increase in serum TG level (Penttinen, 1995). However, the exact mechanisms remain to be elucidated.

Although these studies report the association between low serum lipids level and depression or suicidality, however, conflicting findings have been reported. For instance, some studies fail to identify any correlation between serum lipids level and depression (Deisenhammer et al., 2004; Tanskanen et al., 2000; McCallum et al., 1994). Other studies did not find any association between suicidality and lipid profile in major depressive disorder (Bartoli et al., 2017a; Capuzzi et al., 2018). Other reports suggest that depression is associated with higher serum cholesterol level (Moreira et al., 2017; Nakao & Yano, 2004; Ledochowski et al., 2003). These discrepancies across studies might be attributed to the fact that these studies usually do not distinguish between bipolar and unipolar depressed patients. Clinically, bipolar depression is frequently misdiagnosed as unipolar depression (Shen et al., 2018). At present, studies on the comparison of lipid profile between bipolar and unipolar depression are rare. Identifying the differences in lipid levels between unipolar depression and bipolar depression may be helpful for differential diagnosis between them. It should be noted that recent meta-analyses estimated an association between lipid profile and suicide attempts in major depression (Wu et al., 2016), but not in bipolar disorder (Bartoli et al., 2017b). Huang et al. have reported that patients with bipolar depression have state-dependent alterations of lipid profiles (Huang et al., 2018). The mean cholesterol level was significantly lower in acute mania than in acute depression. The patients in acute bipolar mania have the lowest rate of dyslipidemia (hypertriglyceridemia or low high-density lipoprotein cholesterol) among all states of bipolar depression.

Anhedonia is defined as reduced ability to experience pleasure (Gorwood, 2008). Accumulating evidence has shown that inflammation mechanism/blood lipids participate in the pathophysiological process of anhedonia (Kuehner, 2003; Serafini et al., 2018; Bauer et al., 2008; Wysokiński et al., 2015; Shomaker et al., 2011). According to the Diagnostic and Statistical Manual of Mental Disorders V (DSM-5), anhedonia is one of the main symptoms in the diagnostic criteria for unipolar depression (Gorwood, 2008). Given that lipid abnormality is associated with depression, anhedonia may have an effect on lipid level in depressed patients. In addition to lipid profile, glycemia and thyroid function marker have been shown to be associated with depression or suicidality (Feng et al., 2019; Pompili et al., 2012; Moreira, 2019). For instance, FT3 and FT4 could be used as markers for severity and symptoms of untreated first-episode patients with unipolar depression (Feng et al., 2019). Pompili et al. have reported that suicidal attempters are 2.27 times less likely to have higher FT3 level as compared with non-attempters (Pompili et al., 2012). It has been shown that patient with depression and anhedonia have significant higher level of glucose as compared with healthy control (Moreira, 2019). The purpose of the present study was to investigate the differences in the serum levels of glucose, lipid, and thyroid function markers between unipolar and bipolar depressed patients, as well as the effect of anhedonia and suicidal thoughts on the levels of these biochemical parameters.

## Methods

### Study subjects

A total of 287 unmedicated patients admitted to our hospital from January 2016 to December 2017 due to depression were included in this study. Depression was diagnosed by the Mini International Neuropsychiatric Interview Plus (MINI). The level of depression was evaluated by Self-rating Unipolar depression Scale of Zung (SDS) (Z, 1965). Unipolar and bipolar depression was diagnosed by using the DSM-5. Meanwhile, 89 healthy individuals undergoing physical examination were collected as the healthy control group. All subjects had their blood drawn within 24–72 h after definite diagnosis. This study was approved by the Institutional Review Boards of Nanfang Hospital of Southern Medical University. The requirement of patient informed consent was waived in view of the retrospective nature of the study.

### Data collection

Demographic and clinical data were collected from the medical record. Biochemical parameters include serum lipids (triglycerides, cholesterol, HDL, LDL, very low-density lipoprotein cholesterol [VLDL]), glucose, thyroid function parameters (thyroxine [T4]), free T4 [FT4], low triiodothyronine [T3], free T3 (FT3), thyroid-stimulating hormone [TSH]), prolactin and cortisol were collected from medical record. BMI = weight/height^2^(kg/cm^2^). Insulin resistance index was calculated by the formula Triglycerides/HDL (Ray et al., 2012).

### Measurement of anhedonia and suicidal thoughts

Anhedonia was determined using the item 32 of Symptom Checklist (SCL-90). It is a self-reported of feeling *no interest in things* of 5 grades, and the score greater than or equal to 3 was defined as anhedonia. Suicide ideation was assessed by item 15 of Symptom Checklist-90 (SCL-90). It is a self-reported thought of death or dying of 5 grades, and the score greater than or equal to 3 was defined as suicide ideation.

### Statistical analysis

Continuous variables were presented as the mean ± standard deviation (SD) and were compared by Student’s independent t-test or one-way ANOVA depends on the number of groups. Fisher’s LSD test was used as a post-hoc comparison after ANOVA reaching significant. Categorical data were indicated by number and percentage (%), then were further compared by Chi-square test or Fisher’s exact test (if any expected value lower than 5 was observed). Univariate (crude) and multivariate (adjusted) logistic regression were used to estimate the odds ratio (and its 95% CI) of independent variables to diagnosis or psychological index with dichotomous results (bipolar depression/unipolar depression; with or without anhedonia; with or without Suicidal thoughts). Point-biserial correlation coefficient was used between age (continuous) and dichotomous depression types. ROC analysis was used to investigate the diagnostic effectiveness of continuous variables to dichotomous results, and the AUC, sensitivity, and specificity were reported. All analyses were performed using IBM SPSS Version 20 (SPSS Statistics V20, IBM Corporation, Somers, New York).

## Results

### Participant’s characteristics

A total of 376 subjects (116 males and 260 females, mean age = 40.13 ± 16.058 years) were included in this study. There were 89 cases of healthy control (control group), 92 cases of bipolar depression (bipolar group) and 195 cases of unipolar depression (unipolar group). There were significant differences in the patient’s gender, age, and baseline SBP between the unipolar and bipolar groups (all *P* < 0.05, Table 1).

**Table 1:** Patient’s demographic and clinical characteristics

					P	
Parameters	Control (*n* = 89)	Bipolar depression (*n* = 92)	Unipolar depression (*n* = 195)	Total (*n* = 376)	Overall	Bipolar depression vs. Unipolar depression
Gender					0.002	< 0.001
Male	25 (28.09)	42 (45.65)	49 (25.13)	116 (30.85)		
Female	64 (71.91)	50 (54.35)	146 (74.87)	260 (69.15)		
Age, years	47.34 ± 13.05	24.76 ± 12.75*	44.10 ± 13.94	40.13 ± 16.08	< 0.001	< 0.001
BMI, kg/m2	21.88 ± 3.14	21.38 ± 4.13	21.87 ± 2.92	21.76 ± 3.30	0.474	0.249
SBP, mmHg	121.82 ± 16.08	118.36 ± 13.00	122.69 ± 16.68	121.43 ± 15.77	0.098	0.033
DBP, mmHg	76.36 ± 10.27	73.91 ± 11.41	76.47 ± 10.99	75.82 ± 10.96	0.168	0.070
*Biochemical parameters*						
Glucose	4.86 ± 0.58	4.91 ± 1.14	5.11 ± 1.36	5.00 ± 1.17	0.171	0.184
Triglycerides	1.29 ± 0.60	1.05 ± 0.63	1.46 ± 1.21	1.32 ± 0.99	0.004	0.001
Cholesterol	4.65 ± 0.87	4.27 ± 0.98*	4.75 ± 1.06	4.61 ± 1.02	< 0.001	< 0.001
HDL	1.17 ± 0.28	1.18 ± 0.29	1.21 ± 0.30	1.19 ± 0.29	0.508	0.400
LDL	2.89 ± 0.75	2.59 ± 0.78*	2.90 ± 0.84	2.82 ± 0.81	0.008	0.003
VLDL	0.60 ± 0.23	0.50 ± 0.27*	0.64 ± 0.35	0.60 ± 0.31	0.002	< 0.001
Prolactin	33.26 ± 29.65	51.20 ± 63.75*	38.73 ± 35.95	40.67 ± 44.08	0.021	0.029
T4	7.62 ± 1.55	7.04 ± 1.69*	7.28 ± 1.58	7.30 ± 1.61	0.071	0.266
FT4	1.12 ± 0.18	1.19 ± 0.21*	1.17 ± 0.19	1.16 ± 0.19	0.048	0.293
T3	0.93 ± 0.19	0.94 ± 0.20	0.88 ± 0.18*	0.91 ± 0.19	0.022	0.017
FT3	3.03 ± 0.40	3.28 ± 0.49*	2.96 ± 0.40	3.06 ± 0.45	< 0.001	< 0.001
TSH	1.98 ± 1.63	1.99 ± 1.47	2.23 ± 3.47	2.11 ± 2.73	0.698	0.493
Cortisol	15.50 ± 6.39	17.11 ± 8.09	17.68 ± 7.78*	17.02 ± 7.59	0.104	0.562
Insulin resistance	1.23 ± 0.79	0.99 ± 0.77	1.40 ± 1.61	1.26 ± 1.29	0.043	0.012
*Psychological index*						
Anhedonia					< 0.001	0.003
No	62 (72.94)	34 (38.20)	40 (21.28)	136 (37.57)		
Yes	23 (27.06)	55 (61.80)	148 (78.72)	226 (62.43)		
Suicidal thoughts					< 0.001	0.143
No	78 (91.76)	51 (57.30)	90 (47.87)	219 (60.50)		
Yes	7 (8.24)	38 (42.70)	98 (52.13)	143 (39.50)		

*, *P* < 0.05 compared to control group in post-hoc comparisons

*HDL* high-density lipoprotein cholesterol; *LDL* low-density lipoprotein cholesterol, *VLDL* very low-density lipoprotein cholesterol; *T3* triiodothyronine; *FT3* free T3; FT4, free T4; *TSH* thyroid-stimulating hormone

### Comparison of lipid levels and thyroid function parameters

The lipid levels and other biochemical parameters were compared among 3 groups (overall comparison) and between bipolar and unipolar groups. It was found that Triglycerides, Cholesterol, LDL, VLDL, prolactin, T3, FT3, and insulin resistance were significantly different in both overall comparisons and between two depression groups (all *P* < 0.05). The FT4 level was significantly different in overall comparison (*P* < 0.05). Meanwhile, compared to the control group, the bipolar group had significantly lower levels of cholesterol, LDL, VLDL, T4 but higher levels of prolactin, FT3, FT4. The unipolar group had significantly lower T3 but higher cortisol level than the control group.

### Anhedonia and suicidal thoughts

Participant’s psychological index including anhedonia and suicidal thoughts (both were assessed by SCL90) were compared. As shown in Table 1. both bipolar and unipolar depression groups had a significantly higher incidence of anhedonia than the control group (all *P* < 0.001). The incidence of anhedonia was higher in the unipolar than in the bipolar group (*P* = 0.003).

### Subgroup analysis stratified by anhedonia and suicidal thoughts

To investigate the lipid levels and other biochemical parameters in the depressed patients with or without anhedonia/suicidal thoughts, subgroup analysis stratified by anhedonia and suicidal thoughts was performed. As shown in Table 2, the anhedonia group had significantly lower LDL level than the non-anhedonia (*P* = 0.047). The suicidal thoughts group had significantly higher levels of Cholesterol and HDL than the non-suicidal thoughts (both *P* < 0.05).

**Table 2:** Grouped comparisons in each psychological index

	Anhedonia				Suicidal thoughts			
Parameters	No	Yes	P		No	Yes	P	
Age, years	35.61 ± 16.37	38.78 ± 16.24	0.152		37.13 ± 17.04	38.76 ± 15.53	0.406	
BMI, kg/m2	22.11 ± 3.86	21.59 ± 3.18	0.257		21.92 ± 3.38	21.52 ± 3.37	0.327	
SBP, mmHg	123.38 ± 18.09	120.39 ± 14.57	0.161		121.35 ± 14.77	121.02 ± 16.46	0.864	
DBP, mmHg	76.36 ± 12.83	75.46 ± 10.60	0.560		75.55 ± 10.44	75.85 ± 12.00	0.821	
Glucose	5.10 ± 1.25	5.03 ± 1.35	0.705		5.11 ± 1.09	4.98 ± 1.52	0.409	
Triglycerides	1.37 ± 1.20	1.32 ± 1.05	0.746		1.36 ± 1.24	1.32 ± 0.92	0.752	
Cholesterol	4.76 ± 1.21	4.54 ± 0.98	0.129		4.47 ± 1.15	4.73 ± 0.92	0.035	
HDL	1.16 ± 0.27	1.20 ± 0.30	0.298		1.13 ± 0.28	1.26 ± 0.29	< 0.001	
LDL	2.97 ± 0.95	2.74 ± 0.77	0.047		2.74 ± 0.90	2.87 ± 0.73	0.202	
VLDL	0.63 ± 0.39	0.59 ± 0.31	0.420		0.59 ± 0.39	0.61 ± 0.27	0.713	
Prolactin	39.17 ± 39.56	44.68 ± 50.78	0.414		39.15 ± 36.24	47.30 ± 57.42	0.174	
T4	7.25 ± 1.51	7.18 ± 1.67	0.779		7.02 ± 1.41	7.38 ± 1.81	0.079	
FT4	1.19 ± 0.21	1.17 ± 0.19	0.513		1.16 ± 0.18	1.19 ± 0.21	0.229	
T3	0.92 ± 0.18	0.89 ± 0.20	0.274		0.92 ± 0.20	0.88 ± 0.19	0.147	
FT3	3.14 ± 0.42	3.04 ± 0.47	0.148		3.12 ± 0.48	3.02 ± 0.42	0.077	
TSH	1.99 ± 1.61	2.23 ± 3.35	0.570		2.14 ± 1.87	2.20 ± 3.81	0.884	
Cortisol	18.13 ± 7.35	17.02 ± 7.91	0.307		17.39 ± 7.64	17.25 ± 7.92	0.885	
Insulin resistance	1.38 ± 1.76	1.25 ± 1.30	0.505		1.40 ± 1.73	1.17 ± 1.03	0.176	

*HDL* high-density lipoprotein cholesterol; *LDL* low-density lipoprotein cholesterol, *VLDL* very low-density lipoprotein cholesterol; *T3* triiodothyronine; *FT3* free T3; FT4, free T4; *TSH* thyroid-stimulating hormone

### The diagnostic effectiveness of independent variables to each index

To further confirm the above findings of different levels of biochemical parameters between groups, logistic regression, and ROC analysis were performed in all depressed patients (*n* = 287). As shown in Table 3. unipolar patients were more likely to have higher female proportion, higher levels of SBP, triglycerides, Cholesterol, LDL, VLDL, and insulin resistance but lower levels of T3 and FT3 than the bipolar patients (all *P* < 0.05). However, ROC analysis showed that the most AUCs ranged from 0.55 to 0.68, indicating medium diagnostic effectiveness.

**Table 3:** Logistic regression and ROC analysis results

	Logistic regression (Crude)		Logistic regression (Adjusted ^d^)		ROC analysis	
Parameters	OR (95% CI)	P	OR (95% CI)	P	AUC (95% CI)	P
*Bipolar depression (as reference)* vs. *Unipolar depression* ^a^						
Gender						
Male	ref.	–	ref.	–	–	–
Female	2.50 (1.48–4.22)	< 0.001	1.48 (0.75–2.91)	0.260	–	–
Age, year	1.10 (1.08–1.13)	< 0.001	1.10 (1.07–1.13)	< 0.001	–	–
SBP, mmHg	1.02 (1.00–1.04)	0.033	1.00 (0.97–1.02)	0.824	0.551 (0.479–0.623)	0.177
Triglycerides	1.82 (1.21–2.73)	0.004	1.28 (0.84–1.95)	0.249	0.620 (0.550–0.689)	0.001
Cholesterol	1.60 (1.23–2.09)	< 0.001	1.31 (0.28–6.05)	0.732	0.633 (0.563–0.703)	< 0.001
LDL	1.64 (1.17–2.29)	0.004	0.95 (0.62–1.45)	0.797	0.611 (0.540–0.682)	0.003
VLDL	4.72 (1.85–12.05)	0.001	1.40 (0.30–6.47)	0.666	0.626 (0.557–0.696)	< 0.001
Prolactin	0.99 (0.99–1.00)	0.060	0.99 (0.99–1.00)	0.092	0.546 (0.470–0.622)	0.238
T3	0.19 (0.05–0.76)	0.019	0.62 (0.11–3.36)	0.577	0.598 (0.522–0.675)	0.012
FT3	0.19 (0.10–0.37)	< 0.001	0.73 (0.32–1.66)	0.453	0.685 (0.613–0.758)	< 0.001
Insulin resistance	1.41 (1.04–1.92)	0.028	0.51 (0.18–1.47)	0.214	0.588 (0.517–0.659)	0.019
*Anhedonia* ^b^						
LDL	0.72 (0.52–1.00)	0.049	0.64 (0.45–0.91)	0.013	0.560 (0.482–0.638)	0.129
*Suicidal thoughts* ^c^						
Cholesterol	1.28 (1.02–1.62)	0.037	1.30 (1.01–1.67)	0.043	0.589 (0.522–0.657)	0.011
HDL	4.71 (1.97–11.27)	< 0.001	3.81 (1.55–9.33)	0.003	0.636 (0.571–0.702)	< 0.001

^a^dependent variable was diagnosis, including only bipolar depression and unipolar depression patients where bipolar depression as compared reference

^b^ dependent variable was anhedonia (no or yes), ‘no’ as reference

^c^ dependent variable was Suicidal thoughts (no or yes), ‘no’ as reference

^d^ Depression model was adjusted with patient’s age, gender, triglyceridess, HDL and LDL cholesterol; anhedonia and suicidal thoughts models was adjusted with patient’s age and gender

*HDL* high-density lipoprotein cholesterol; *LDL* low-density lipoprotein cholesterol, *VLDL* very low-density lipoprotein cholesterol; *T3* triiodothyronine; FT3, free T3; FT4, free T4; *TSH* thyroid-stimulating hormone

In the subgroup of anhedonia, patients with anhedonia were more likely to have lower LDL than those without anhedonia (*P* = 0.049, Table 3), but the result of ROC analysis did not significant (*P* = 0.129, Table 3). As for the subgroup of suicidal thoughts, patients with suicidal thoughts group were more likely to have higher Cholesterol and HDL than those without suicidal thoughts (both *P* < 0.05, Table 3). The AUCs were 0.589 and 0.636, respectively (both *P* < 0.05, Table 3).

Multivariate (adjusted) logistic regression adjusting for patient’s age, gender, triglyceridess, HDL and LDL cholesterol levels was conducted to investigate the results between independent variable and depression diagnosis (bipolar vs. unipolar depression). However, in multivariate results, age was the only variable significantly different between the bipolar and unipolar depressions (Table 3). For the multivariate models of anhedonia and suicidal thoughts, patient’s age and gender were adjusted. Similar to the univariate results, significant associations were found between: (1) LDL and anhedonia; (2) cholesterol, HDL and suicidal thoughts (all *P* < 0.05).

## Discussion

In this study, we investigated the differences in the serum levels of glucose, lipid, and thyroid function markers between unipolar and bipolar depressed patients, as well as the effect of anhedonia and suicidal thoughts on the levels of these biochemical parameters. The results showed that the bipolar group had significantly lower lipid levels (including triglycerides, cholesterol, LDL, VLDL) and insulin resistance index but higher levels of T3 and FT3 as well as a higher incidence of anhedonia as compared with the unipolar group. Depressed patients with anhedonia had significantly higher LDL level than those without anhedonia. Depressed patients with suicidal thoughts had higher cholesterol and HDL levels. The differences in the serum lipid and T3, FT3 levels between the unipolar and bipolar groups were confirmed by Logistic regression analysis. The differences in LDL and cholesterol/HDL in subgroup analysis stratified by anhedonia and suicidal thoughts were also confirmed by Logistic regression analysis. ROC analysis showed that AUC ranged from 0.546 to 0.685. Taken together, these results suggested that triglycerides, cholesterol, LDL, VLDL T3, FT3 levels were significantly different between unipolar and bipolar depression.

The AUC value of 0.5 to 0.7 means moderate diagnostic effectiveness, indicating the association between independent continuous variable and dichotomous outcome. However, an AUC value < 0.8 may imply insufficient diagnostic effectiveness in clinical practice. Small sample size and unexpected confounding sources also influence the estimation of AUC. Future works of larger sample size study and the control of confounding are needed to verify the effectiveness of these independent variables. The assessment of anhedonia and suicidal ideation by SCL-90 item 32 and 15 was based on previous studies (Tang et al., 1999; Valach, 2018) and our consideration. According to Tang et al.’s study (Tang et al., 1999), the items of SCL-90 corresponding to depression symptoms and could be used to discriminate depression subjects from healthy controls were item 5, 14, 15, 20, 22, 26, 29, 30, 31, 32, 54, 71 and 79. For suicidal ideation, the description of item 15 (‘Thoughts of ending own life’) directly indicates the concept of suicidal ideation, which has been used to assess suicidal ideation (Valach, 2018). However, the item corresponding to anhedonia was selected by our own judgment where the description of item 32 (‘Feeling no interest in things’) was the most closed concept to anhedonia. Therefore, we chose these two item scores to represent these two symptoms.

We noticed a remarkable female preponderance (74.87% of females) in our unipolar depressed patients. The gender difference in unipolar depression has been widely reported (Kuehner, 2003; Sprock & Yoder, 2007; Wolk & Weissman, 1995). At present, the mechanism underlying female preponderance in unipolar depression remains not fully understood, although several possible explanations have been proposed, such as genetics, sex hormones, neuropsychological factors, gender role and psychosocial factors (Kuehner, 2003). However, the exact mechanism remains to be elucidated. Our cohort showed that the mean age was significantly younger in the bipolar group than in the unipolar group (24.76 ± 12.75 vs. 44.10 ± 13.94, *P* < 0.001). This phenomenon should be attributed to the fact that bipolar depressed patients have a younger age at illness onset than the unipolar depressed patients (Serafini et al., 2018; Leonpacher et al., 2015). Our multivariate results showed that age was the only significant variable, which might be attributed to nature essential distribution of age between bipolar and unipolar depressions. Studies have revealed that the onset age of bipolar depression is usually earlier than unipolar depression (Serafini et al., 2018; Leonpacher et al., 2015; Nisha et al., 2015; Shippee et al., 2011; Yatham et al., 2018). The strong correlation between age and depression type (point-biserial correlation coefficient *r* = 0.56, *P* < 0.001) occupied the most variance of explanatory variables, making other variables difficult to be significant in the multivariate logistic regression analysis. Therefore, the results of the multivariate model seemed to be not credible, and the results of univariate (crude) logistic regression and ROC analysis should be considered.

Studies on directly comparing the serum lipid profiles between unipolar and bipolar depression are extremely limited (Wysokiński et al., 2015). Our results showed significant differences in the triglycerides, cholesterol, LDL, VLDL levels between two depression groups. In addition, the differences were confirmed by Logistic regression analysis. The total SDS score was not significantly different between unipolar and bipolar patients (62.53 ± 14.71 vs. 65.30 ± 16.27, *P* = 0.168). These results indicated that although the two forms of depressed patients had a comparable level of depression, there was still a difference in lipid profile between groups. However, the trend and types of these metabolic dysfunctions are not exactly consistent with a previous study (Wysokiński et al., 2015). The inconsistent observations may be due to the fact that we did not distinguish among different disease stages in our bipolar patients. Mixed stages of bipolar depression may affect the analysis results. On the other hand, the young age of our bipolar patients may also contribute to the discrepancy.

It has been found that the abnormal change in insulin resistance is earlier than the changes in the levels of blood glucose and triglycerides (Deisenhammer et al., 2004). In the pathogenesis of insulin resistance and metabolic syndrome, abnormalities of glucose and lipid metabolism can mutually affect and promote (Deisenhammer et al., 2004). Depression has been shown to increase the risk of progressive insulin resistance (Shomaker et al., 2011). Our study demonstrated that unipolar depressed patients had a higher level of insulin resistance than bipolar patients. However, the underlying mechanism is still to be elucidated.

In addition to lipid profile, thyroid function has also been found to be related to mood disorder, including depression (Bauer et al., 2008). Both hyperthyroidism and hypothyroidism can cause mood abnormalities (Hage & Azar, 2012). The fluctuations in serum levels of thyroid hormones have been reported in depressed patients, including elevated T4 level, T3, elevated 3,3′,5′-Triiodothyronine (rT3) (Chopra et al., 1990; Premachandra et al., 2006; Kirkegaard et al., 1990; Van de Ven et al., 2012). In addition, TSH level has been shown to be associated with depression severity (Bauer et al., 2008), and hyperthyroidism can elevate the risk for bipolar depression (Hu et al., 2013). Supporting these observations, our results showed that T3 and FT3 levels were higher in the bipolar group than in the unipolar group, implying that T3 might be a potential marker for distinguishing bipolar from unipolar depression. Nevertheless, the mechanism is needed to be investigated.

In our subgroup analysis stratified by anhedonia, depressed patients (including unipolar and bipolar) with anhedonia had significantly lower LDL level than those without anhedonia. Our result is inconsistent with Moreira et al. recent report that subjects with unipolar depression and anhedonia have a significant increase in LDL level (Moreira, 2019). The difference in the subject’s composition may contribute to the conflicting findings.

Our subgroup analysis stratified by suicidal thoughts showed that depressed patients with suicidal thoughts had significantly higher cholesterol and HDL levels than those without suicidal thoughts. Our finding is similar to the previous study that recent suicide attempters had a lower serum triglycerides and a higher HDL than those without suicide attempt in depressed patients (Baek et al., 2014). However, several studies report that low cholesterol level increases suicide risk (Kunugi et al., 1997; Wu et al., 2016; Sullivan et al., 1994), which is not in line with our observations. The discrepancies across studies might be the differences in the measurement of suicidal thoughts.

There are still some limitations of this study. First, this was a retrospective study, and some assessments cannot be conducted to obtain data. For example, the presence of anhedonia is typically screened by the Snaith-Hamilton Pleasure Scale (SHAPS) (Snaith et al., 1995). However, since this was a retrospective analysis of the medical record, it can only be replaced by the medical record data (item 32 of SCL-90). In addition, we did not collect the information about possible confounders for analysis, such as smoke, exercise, substance use disorder. Furthermore, the sample size was still relatively small and unequal between the two groups. In the future, a prospective trial with a large sample size should be performed to further validate the findings of this study.

## Conclusions

In summary, our findings demonstrated that there were significant differences in the levels of some lipid (triglycerides, cholesterol, LDL, VLDL) and T3 and FT3 between unipolar and bipolar depression, which might have the potential to develop as the markers for distinguishing bipolar from unipolar depression. Patients with anhedonia had lower LDL level, while patients with suicidal thoughts had higher levels of cholesterol and HDL as compared with the corresponding control groups.

## Data Availability

All the data and material were presented in the main paper.

## Abbreviations

DSM-5: Diagnostic and Statistical Manual of Mental Disorders V
HDL: High-density lipoprotein cholesterol
LDL: Low-density lipoprotein cholesterol
MINI: Mini International Neuropsychiatric Interview Plus
SD: Standard deviation
SHAPS: Snaith-Hamilton Pleasure Scale

## References

[CR1] Ainiyet B, Rybakowski JK (2014). Suicidal behaviour and lipid levels in unipolar and bipolar depression. Acta Neuropsychiatr.

[CR2] Baek JH, Kang ES, Fava M, Mischoulon D, Nierenberg AA, Yu BH (2014). Serum lipids, recent suicide attempt and recent suicide status in patients with major depressive disorder. Prog Neuro-Psychopharmacology Biol Psychiatry.

[CR3] Bartoli F, Crocamo C, Dakanalis A, Riboldi I, Miotto A, Brosio E (2017). Association between total serum cholesterol and suicide attempts in subjects with major depressive disorder: exploring the role of clinical and biochemical confounding factors. Clin Biochem.

[CR4] Bartoli F, Di Brita C, Crocamo C, Clerici M, Carrà G (2017). Lipid profile and suicide attempt in bipolar disorder: a meta-analysis of published and unpublished data. Prog Neuro-Psychopharmacol Biol Psychiatry.

[CR5] Bauer M, Goetz T, Glenn T, Whybrow PC (2008). The thyroid-brain interaction in thyroid disorders and mood disorders. J Neuroendocrinol.

[CR6] Capuzzi E, Bartoli F, Crocamo C, Malerba MR, Clerici M, Carrà G (2018). Recent suicide attempts and serum lipid profile in subjects with mental disorders: a cross-sectional study. Psychiatry Res.

[CR7] Chopra IJ, Solomon DH, Huang TS (1990). Serum thyrotropin in hospitalized psychiatric patients: evidence for hyperthyrotropinemia as measured by an ultrasensitive thyrotropin assay. Metabolism..

[CR8] Deisenhammer EA, Kramer-Reinstadler K, Liensberger D, Kemmler G, Hinterhuber H, Fleischhacker WW (2004). No evidence for an association between serum cholesterol and the course of depression and suicidality. Psychiatry Res.

[CR9] Feng GH, Kang CY, Yuan J, Zhang Y, Wei YJ, Xu L (2019). Neuroendocrine abnormalities associated with untreated first episode patients with major depressive disorder and bipolar disorder. Psychoneuroendocrinology.

[CR10] Gorwood P (2008). Neurobiological mechanisms of anhedonia. Dialogues Clin Neurosci.

[CR11] Hage MP, Azar ST (2012). The link between thyroid function and depression. J Thyroid Res.

[CR12] Hawton K, Cowen P, Owens D, Bond A, Elliott M (1993). Low serum cholesterol and suicide. Br J Psychiatry.

[CR13] Hu LY, Shen CC, Hu YW, Chen MH, Tsai CF, Chiang HL (2013). Hyperthyroidism and risk for bipolar disorders: a Nationwide population-based study. PLoS One.

[CR14] Huang YJ, Tsai SY, Chung KH, Chen PH, Huang SH, Kuo CJ (2018). State-dependent alterations of lipid profiles in patients with bipolar disorder. Int J Psychiatry Med.

[CR15] Kirkegaard C, Kørner A, Faber J (1990). Increased production of thyroxine and inappropriately elevated serum thyrotropin in levels in endogenous depression. Biol Psychiatry.

[CR16] Kuehner C (2003). Gender differences in unipolar depression: an update of epidemiological findings and possible explanations. Acta Psychiatr Scand.

[CR17] Kunugi H, Takei N, Aoki H, Nanko S (1997). Low serum cholesterol in suicide attempters. Biol Psychiatry.

[CR18] Ledochowski M, Murr C, Sperner-Unterweger B, Neurauter G, Fuchs D (2003). Association between increased serum cholesterol and signs of depressive mood. Clin Chem Lab Med.

[CR19] Leonpacher AK, Liebers D, Pirooznia M, Jancic D, Mackinnon DF, Mondimore FM (2015). Distinguishing bipolar from unipolar depression: the importance of clinical symptoms and illness features. Psychol Med.

[CR20] Maes M, Smith R, Christophe A, Vandoolaeghe E, Van Gastel A, Neels H (1997). Lower serum high-density lipoprotein cholesterol (HDL-C) in major depression and in depressed men with serious suicidal attempts: relationship with immune-inflammatory markers. Acta Psychiatr Scand.

[CR21] McCallum J, Simons J, Simons L, Friedlander Y (1994). Low serum cholesterol is not associated with depression in the elderly: data from an Australian community study. Aust NZ J Med.

[CR22] Moreira FP (2019). Jansen K, Cardoso T de a, Mondin TC, Vieira IS, Magalhães PV da S, et al. metabolic syndrome, depression and anhedonia among young adults. Psychiatry Res.

[CR23] Moreira FP, Jansen K, Cardoso TA, Mondin TC, PVDS M, Kapczinski F (2017). Metabolic syndrome in subjects with bipolar disorder and major depressive disorder in a current depressive episode: Population-based study: Metabolic syndrome in current depressive episode. J Psychiatr Res.

[CR24] Morgan RE, Palinkas LA, Barrett-Connor EL, Wingard DL (1993). Plasma cholesterol and depressive symptoms in older men. Lancet..

[CR25] Nakao M, Yano E (2004). Relationship between major depression and high serum cholesterol in Japanese men. Tohoku J Exp Med.

[CR26] Nisha A, Sathesh V, Punnoose VP, Varghese PJ (2015). A comparative study on psycho-socio-demographic and clinical profile of patients with bipolar versus unipolar depression. Indian J Psychiatry.

[CR27] Olusi SO, Fido AA (1996). Serum lipid concentrations in patients with major depressive disorder. Biol Psychiatry.

[CR28] Ong KL, Morris MJ, McClelland RL, Maniam J, Allison MA, Rye KA (2016). Lipids, lipoprotein distribution and depressive symptoms: the multi-ethnic study of atherosclerosis. Transl Psychiatry.

[CR29] Penttinen J (1995). Hypothesis: low serum cholesterol, suicide, and lnterleukin-2. Am J Epidemiol.

[CR30] Pompili M, Gibiino S, Innamorati M, Serafini G, Del Casale A, De Risio L (2012). Prolactin and thyroid hormone levels are associated with suicide attempts in psychiatric patients. Psychiatry Res.

[CR31] Premachandra BN, Kabir MA, Williams IK (2006). Low T3 syndrome in psychiatric depression. J Endocrinol Investig.

[CR32] Ray S, Bairagi AK, Guha S, Ganguly S, Ray D, Basu AK (2012). A simple way to identify insulin resistance in non-diabetic acute coronary syndrome patients with impaired fasting glucose. Indian J Endocrinol Metab.

[CR33] Serafini G, Lamis D, Canepa G, Aguglia A, Monacelli F, Pardini M (2018). Differential clinical characteristics and possible predictors of bipolarity in a sample of unipolar and bipolar inpatients. Psychiatry Res.

[CR34] Shen H, Zhang L, Xu C, Zhu J, Chen M, Fang Y (2018). Analysis of misdiagnosis of bipolar disorder in an outpatient setting. Shanghai Arch Psychiatry.

[CR35] Shippee ND, Shah ND, Williams MD, Moriarty JP, Frye MA, Ziegenfuss JY (2011). Differences in demographic composition and in work, social, and functional limitations among the populations with unipolar depression and bipolar disorder: results from a nationally representative sample. Health Qual Life Outcomes.

[CR36] Shomaker LB, Tanofsky-Kraff M, Stern EA, Miller R, Zocca JM, Field SE (2011). Longitudinal study of depressive symptoms and progression of insulin resistance in youth at risk for adult obesity. Diabetes Care.

[CR37] Snaith RP, Hamilton M, Morley S, Humayan A, Hargreaves D, Trigwell P (1995). A scale for the assessment of hedonic tone. The Snaith-Hamilton pleasure scale. Br J Psychiatry.

[CR38] Sprock J, Yoder CY (2007). Women and depression: an update on the report of the APA task force. Sex Roles.

[CR39] Steegmans PH, Fekkes D, Hoes AW, Bak AA, van der Does E, Grobbee DE (1996). Low serum cholesterol concentration and serotonin metabolism in men. BMJ.

[CR40] Sullivan PF, Joyce PR, Bulik CM, Mulder RT, Oakley-Browne M (1994). Total cholesterol and suicidality in depression. Biol Psychiatry.

[CR41] Tang Q, Cheng Z, Yuan A, Deng Y (1999). The use and reanalysis of SCL-90 in China. Chinese J Clin Psychol.

[CR42] Tanskanen A, Tuomilehto J, Viinamäki H (2000). Cholesterol, depression and suicide. Br J Psychiatry.

[CR43] Tedders SH, Fokong KD, McKenzie LE, Wesley C, Yu L, Zhang J (2011). Low cholesterol is associated with depression among US household population. J Affect Disord.

[CR44] Valach L (2018). SCL-90-R and suicide ideation in torture and war survivors receiving psychotherapy. J Cogn Behav Ther.

[CR45] Van de Ven AC, Muntjewerff JW, Netea-Maier RT, de Vegt F, Ross HA, Sweep FC (2012). Association between thyroid function, thyroid autoimmunity, and state and trait factors of depression. Acta Psychiatr Scand.

[CR46] van Reedt Dortland AK, Giltay EJ, van Veen T, van Pelt J, Zitman FG, Penninx BW (2010). Associations between serum lipids and major depressive disorder. J Clin Psychiatry.

[CR47] Wolk SI, Weissman MM (1995). Women and depression: an update. Am Psychiatr Press Rev Psychiatry.

[CR48] Wu S, Ding Y, Wu F, Xie G, Hou J, Mao P (2016). Serum lipid levels and suicidality: a meta-analysis of 65 epidemiological studies. J Psychiatry Neurosci.

[CR49] Wysokiński A, Strzelecki D, Kłoszewska I (2015). Levels of triglycerides, cholesterol, LDL, HDL and glucose in patients with schizophrenia, unipolar depression and bipolar disorder. Diabetes Metab Syndr.

[CR50] Yatham LN, Kennedy SH, Parikh SV, Schaffer A, Bond DJ, Frey BN (2018). Canadian network for mood and anxiety treatments (CANMAT) and International Society for Bipolar Disorders (ISBD) 2018 guidelines for the management of patients with bipolar disorder. Bipolar Disord.

[CR51] Z WK (1965). A self-rating depression scale. Arch Gen Psychiatry.

[CR52] Zhang J, McKeown RE, Hussey JR, Thompson SJ, Woods JR, Ainsworth BE (2005). Low HDL cholesterol is associated with suicide attempt among young healthy women: the third National Health and nutrition examination survey. J Affect Disord.

